# Physical and dosimetric characteristic of high‐definition multileaf collimator (HDMLC) for SRS and IMRT

**DOI:** 10.1120/jacmp.v12i3.3475

**Published:** 2011-04-14

**Authors:** Dayananda Shamurailatpam Sharma, Prabhakar M Dongre, Vaibav Mhatre, Malhotra Heigrujam

**Affiliations:** ^1^ Department of Radiation Oncology Kokilaben Dhirubhai Ambani Hospital and Medical Research Institute Mumbai 400053 India; ^2^ Department of BioPhysics University of Mumbai Mumbai 400098 India

**Keywords:** MLC, QA, dosimetry, IMRT, SRS

## Abstract

Physical and dosimetric characteristics of HDMLC were studied for SRS6, 6, and 10 MV X‐rays from Novalis Tx. This in‐built tertiary collimator consists of 60 pairs (32×0.25 cm; 26×0.5 cm and 2×0.7 cm) of leaves. Properties of HDMLC studied included alignment, readout and radiation field congruence, radiation penumbra, accuracy and reproducibility of leaf position and gap width, static and dynamic leaf shift, tongue‐and‐groove effect, leaf transmission and leakage, leaf travel speed, and delivery of dynamic conformal arc and IMRT. All tests were performed using a calibrated ionization chamber, film dosimetry and DynaLog file analysis. Alignment of leaves with isocenter plane was better than 0.03 cm at all gantry and collimator positions. The congruence of HDMLC readout and radiation field agreed to within ± 0.03cm for filed sizes ranging from 1×1 to $20\times 20\;{\text{cm}}^{2}$. Mean 80% to 20% penumbra width parallel (perpendicular) to leaf motion was 0.24±0.05(0.21±0.02) cm, 0.37±0.12(0.29±0.07) cm, and 0.51±0.13(0.43±0.07) cm for SRS6, 6, and 10 MV X‐rays, respectively. Circular field penumbra was comparable to corresponding square field. Average penumbra of $1\times 20\;{\text{cm}}^{2}$ field was effectively constant over off‐axis positions of up to 12 cm with mean value of 0.16 (± 0.01)cm at 1.5 cm depth and 0.38 (± 0.04)cm at 10 cm depth. Minimum and maximum effective penumbra along the straight diagonal edge of irregular fields increased from 0.3 and 0.32 cm at 70° steep angle to 0.35 and 0.56 cm at 20° steep angle. Modified Picket Fence test showed average FWHM of 0.18 cm and peak‐to‐peak distance of 1.99 cm for 0.1 cm band and 2 cm interband separation. Dynamic multileaf collimation (DMLC) output factor remained within ± 1% for 6 MV and ± 0.5% for 10 MV X‐rays at all gantry positions, and was reproducible within ± 0.5% over a period of 14 months. The static leaf shift was 0.03 cm for all energies, while dynamic leaf shift was 0.044 cm for 10 MV and 0.039 cm for both SRS6 and 6 MV X‐rays. The dose depression and corresponding tongue‐and‐groove size were 24% and 0.17 cm for 6 MV and 19% and 0.20 cm for 10 MV X‐rays. Average transmission through HDMLC was 1.09%, 1.14% and 1.34% for SRS6, 6 and 10 MV X‐rays. Analysis of DynaLog files for leaf speed test in arc dynamic mode, delivery test of dynamic conformal arc, and step‐and‐shoot and sliding window IMRT showed at least 95% or more of the error counts had misplacements < 0.2cm, with maximum root mean square (RMS) error value calculated at 0.13 cm. Accurate and reproducible leaf position and gap width, and less leakage and small consistent penumbra over the fields demonstrate HDMLC suitable for high‐dose resolution SRS and IMRT.

PACS number: 87.56.N‐, 87.55.Qr, 87.50.cm, 87.55.de, 87.53.Ly

## I. INTRODUCTION

The last two decades have witnessed a continuous development in the multileaf collimator (MLC) hardware and controller software technology. At present, all linear accelerator (linac) manufacturers support MLCs for conformal treatment and intensity‐modulated radiotherapy (IMRT).^(^ ^1^ ^–^ ^5^ ^)^ Different types of MLC design, operating limits, commissioning and quality assurance (QA) requirements, and physical characteristics were extensively reported in AAPM Report No 72.^(5)^ The MLCs differ in physical dimension, leaf side and end design, and in the way they integrate into the collimator assembly. These conventional MLCs with relatively large leaf widths – mostly 1 cm at isocenter – resulted in inferior conformation of dose to small targets generally treated with stereotactic radiosurgery (SRS).^(6)^ Micro‐MLC (mMLC) having a thinner projected leaf width of about 0.3 cm was introduced either as an add‐on accessory to the existing linac or in‐built in a dedicated linac for application in SRS.^(^ ^6^ ^–^ ^9^ ^)^ However, a mMLC suffers from a maximum limited field size of about $10\times 10\;{\text{cm}}^{2}$. A new high‐definition multileaf collimator (HDMLC) having thinner leaf width and yet providing relatively large field size was recently introduced to perform high‐precision radiosurgery and IMRT. The accurate delivery of dose from these techniques primarily rely on the physical and dosimetric characteristics of the MLC and also, in part, on the proprietary controller software.^(^ ^2^ ^,^ ^10^ ^–^ ^13^ ^)^ Performance data of commercially available conventional MLCs^(^ ^2^ ^–^ ^4^ ^,^ ^11^ ^–^ ^24^ ^)^ and mMLCs^(^ ^6^ ^–^ ^9^ ^)^ from various manufacturers have been reported separately for application in conformal, IMRT and SRS. In this study, we investigate the physical and dosimetric characteristics of HDMLC controlled by a new software for clinical commissioning of dynamic conformal SRS and IMRT.

## II. MATERIALS AND METHODS

**Figure 1:**
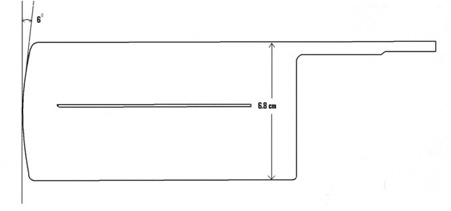
Schematic diagram of a single leaf of HDMLC.

The HDMLC integrated as tertiary collimator in Novalis Tx (Varian Medical System, Palo Alto, CA) consists of 60 pairs (inner 32 pairs of 0.25 cm; outer 26 pairs of 0.5 cm and outermost 2 pairs of 0.7 cm width at isocenter) of tungsten leaves. It is placed with the bottom of the central leaf at 51.4 cm from the target. The inner 32 pairs allow for fine shaping of small irregular target commonly treated with radiosurgery, and can define a square field size of up to $8\times 8\;{\text{cm}}^{2}$. A $22\times 22\;{\text{cm}}^{2}$ field size typically used in clinics can be achieved at isocenter using all 60 leaf pairs. In addition to the leaf width, HDMLC differs from the previous models of Varian MLC mainly in leaf end design and leaf height (Fig. 1), which in turn may affect the dosimetric characteristics. While the controller hardware and general operational concept are similar to the Millennium 120 MLC, HDMLC is controlled by new software (V7.2), which partly determines the performance accuracy of the leaves.

The physical and dosimetric characteristics of HDMLC were studied for SRS6, 6, and 10MV X‐rays from Novalis Tx following previously established methods and recommendations, including the recent AAPM TG Report No 142.^(^ ^2^ ^,^ ^6^ ^–^ ^8^ ^,^ ^11^ ^–^ ^13^ ^,^ ^16^ ^–^ ^25^ ^)^ The SRS6 MV is a third photon energy introduced for application in radiosurgery. It has the same beam quality as 6MV but operates at a much higher dose rate of 1000 MU/min with a maximum field size of $15\times 15\;{\text{cm}}^{2}$. In the subsequent investigations, maximum field size for SRS6 MV is limited to $15\times 15\;{\text{cm}}^{2}$. Properties of HDMLC studied include: a) HDMLC alignment, b) HDMLC readout and radiation field congruence, c) radiation penumbra, d) accuracy and reproducibility of leaf position and gap width, e) static and dynamic leaf shift, f) tongue‐and‐groove effect, g) leaf transmission and leakage, h) leaf travel speed, and i) delivery of dynamic conformal arc and IMRT. Similar to other studies^(^ ^2^ ^,^ ^6^ ^–^ ^8^ ^,^ ^11^ ^–^ ^13^ ^,^ ^16^ ^–^ ^25^ ^)^ all tests were carried out using calibrated 0.65 cc ionization chamber, solid plastic slab (SP34; Scanditronic Wellhoffer, Germany) phantom, $35\times 35\times 40\;{\text{cm}}^{3}$ water phantom, Kodak EDR2 radiotherapy verification film (Eastman Kodak Company, Rochester, NY), Vidar VXR‐16 DosimetryPro Advantage film scanner (Vidar Systems Corp., Herndon, VA) with OmniPro IMRT analysis software (Scanditronic Wellhoffer, Germany). Throughout the measurement, all processed films were scanned using Vidar film scanner at 0.17 mm per pixel resolution and analyzed using optical density to dose calibration films and OmniPro IMRT software (V1.6).

### A. HDMLC alignment

**Figure 2:**
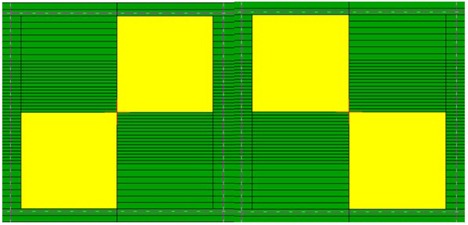
Complementary leaf patters to test HDMLC alignment.

Alignment of the leaves in the direction of leaf motion was initially performed during HDMLC calibration using manufacturer provided alignment bar and calibration software, following recommended procedures.^(26)^ In the first step of the HDMLC calibration, alignment of the leaves was ensured when all leaves lightly touch, without any gap, the alignment bar mounted on the center line of MLC base plate. The second step of calibration was performed without the alignment bar, and proper alignment was ensured when all opposing leaves just touch each other without any gap. Accuracy of HDMLC alignment relative to isocenter plane was verified by exposing Kodak EDR2 verification film placed at isocenter plane using two complementary test patterns, shown in Fig. 2. Effect of gantry and collimator rotation on HDMLC alignment was investigated at gantry 180°, 90°, 270° and collimator at 90° and 270°. For all measurements, film was placed perpendicular to beam central axis. To detect minimum misalignment, films were also exposed with different simulated error ranging from 0.1 cm to 0.01 cm on one side of the HDMLC test pattern shown in Fig. 2.

### B. HDMLC readout and radiation field congruence

EDR2 films kept at isocenter plane with no buildup were exposed using HDMLC‐shaped square field sizes ranging from $1\times 1\;{\text{cm}}^{2}$ to $5\times 5\;{\text{cm}}^{2}$ in step of 1 cm and in 5 cm steps thereafter, up to $20\times 20\;{\text{cm}}^{2}$. The radiation field sizes, measured as the full width at half maximum (FWHM) value of the dose profiles taken parallel and perpendicular to leaf motion of every field, were compared against the corresponding programmed HDMLC field sizes.

### C. Radiation penumbra

EDR2 films placed at $d_{\text{max}}$ (1.5 cm for 6 and SRS6 MV and 3 cm for 10 MV X‐rays) with target to phantom surface distance (TSD) of 100 cm were exposed using the same HDMLC‐shaped square fields used for readout and radiation field congruence test. The average radiation penumbra (80%–20%) parallel and perpendicular to leaf motion was measured from the profiles taken at 0.13 cm offset from the cross‐line. Similarly, the average penumbra parallel to leaf motion was also measured for HDMLC‐shaped circular fields having the same diameter as the side of square fields. Beam penumbra as a function of off‐axis distance was investigated for 6 MV X‐rays using a rectangle field of $1\times 20\;{\text{cm}}^{2}$. Films placed at $d_{\text{max}}$ and 10 cm depth in a solid phantom with TSD of 100 cm were simultaneously irradiated with the center of the field set at 0, 4, 8 and 12 cm off from the central axis. Average penumbra at an off‐axis position was obtained from the multiple dose profiles taken along midleaf pairs of different leaf numbers. The effective beam penumbra,^(21)^ defined as the distance between line joining the peaks of 80% isodose and valleys of 20% isodose, were also investigated for two HDMLC‐shaped irregular fields having different stepping angle ranging from 20° to 70°.^(^ ^6^ ^,^ ^21^ ^,^ ^22^ ^)^ Stepping angle was defined between the straight diagonal edge of the field and the direction of HDMLC motion, and 20° represent the least steeped side of the field in our study.^(22)^ EDR2 films placed at isocenter plane were exposed with appropriate buildup thickness of 1.5 cm for 6, SRS6 and 3 cm for 10 MV X‐rays. Effective penumbra was measured along the straight diagonal edges of every irregular field.^(6)^

### D. Leaf position and gap width in dynamic mode

The accuracy of HDMLC leaf position and gap width in dynamic mode was investigated using modified Picket Fence method.^(11)^ Briefly, an HDMLC test pattern consisting of six narrow bands of 0.1 cm width and 2 cm interband separation was created in dose dynamic mode. EDR2 film kept at isocenter plane was exposed using this test field. Dose profiles were measured at the midleaf position and along the leaf motion at different arbitrarily chosen leaf numbers. To spot minimum detectable error of leaf gap in dynamic mode, modified Picket Fence test was repeated with simulated errors of 0.01, 0.02, 0.05 and 0.1 cm in different bands.

In another test, a static jaw‐defined field of $10\times 10\;{\text{cm}}^{2}$ was simulated in dose dynamic mode by sweeping uniform fields of 0.2×10,0.5×10 and $1\times 10\;{\text{cm}}^{2}$. Dose from these uniform dynamic MLC (DMLC) fields were measured in air for 6 and 10 MV X‐rays at 400 MU/min dose rate using a 0.65 cc ionization chamber positioned at isocenter with appropriate buildup thickness.^(^ ^12^ ^,^ ^13^ ^)^ The integrated dose from each measurement was normalized to the dose measured using static $10\times 10\;{\text{cm}}^{2}$ field under the same condition. This measurement was carried out for different gantry angles of 0°, 90°, 180° and 270°. The long term reproducibility of leaf position and gap width was monitored periodically over a period of 14 months: i) by checking static light field projection of the manufacturer provided MLC test pattern, and ii) repeating measurement of DMLC output factor at least once every month.^(13)^

### E. Static and dynamic leaf shift

Static and dynamic leaf shift describes an effective leaf shift due to the rounded leaf end design of the HDMLC. EDR2 films kept isocentrically at 5 cm depth (TSD=95 cm) in solid water phantom were exposed with SRS6, 6, and 10 MV X‐rays using a filed size of $10\times 10\;{\text{cm}}^{2}$. The dosimetric static leaf shift was measured as the difference between radiation field width defined by rounded leaf end design of HDMLC and corresponding calibration field.

For dynamic leaf shift measurement, a field size of $10\times 10\;{\text{cm}}^{2}$ was simulated in dynamic mode using different sweeping gap width of 0.1, 0.5, 1, 2, 5, 10 cm, respectively. Ionization from these uniform DMLC fields was measured for 400 MU at a dose rate 400 MU/min for 6 and 10 MV X‐rays and 1000 MU at 1000 MU/min for SRS6 MV in a water phantom using a 0.65 cc calibrated ionization chamber positioned at the $d_{\text{max}}$ of the respective energy. The measured dose free from HDMLC leakage was plotted against the sweeping gap width. The effective dynamic leaf shift (δ) was estimated from the straight plot using the relation:

(1) δ=(Intercept/2×slope)

### F. Tongue‐and‐groove effect

The dose depression and tongue‐and‐groove size measurement were performed using previously described double exposure radiographic film technique.^(2)^ EDR2 film kept at $d_{\text{max}}$ isocentrically were first exposed with top half of a $10\times 10\;{\text{cm}}^{2}$ field blocked by HDMLC and bottom half open, followed by bottom half blocked and top half open. The profile measured along the match‐line and perpendicular to leaf motion which, when normalized to the average dose of either half, gives the dose depression due to tongue‐and‐grove design of HDMLC. The FWHM taken from the same profile represents tongue‐and‐groove size.

### G. Leaf transmission and leakage

Average leakage radiation from HDMLC was measured on the beam central axis using 0.65 cc ionization chamber positioned at 5 cm depth in a solid water phantom. First, jaws were set at $10\times 10\;{\text{cm}}^{2}$ and HDMLC leaves were fully closed asymmetrically at 5 cm from the central axis. Secondly, both jaws and leaves were closed asymmetrically at 5 cm off‐axis. Doses from both measurements were normalized to central axis dose of $10\times 10\;{\text{cm}}^{2}$ field, with HDMLC fully open to yield average leakage radiation. Inter‐ and intraleaf transmission were measured for 6 MV X‐rays using EDR2 film placed isocentrically at 1.5 cm depth in a solid water phantom. Film was exposed with jaws set at $10\times 10\;{\text{cm}}^{2}$ and HDMLC leaves fully closed asymmetrically at 5 cm from the central axis. The dose profile along the central axis and perpendicular to leaf motion was normalized to open field dose measured on the same film under the same irradiation geometry. The number of MUs for closed HDMLC and open field were adjusted to produce similar optical density on the film.

### H. Leaf travel speed test

The stability of leaf speed was tested in arc dynamic mode using HDMLC controller‐created MLC DynaLog file.^(^ ^23^ ^,^ ^24^ ^,^ ^27^ ^,^ ^28^ ^)^ This file contains information about the planned versus actual position for all leaves every 50 ms while the beam is on. The software takes data and creates a series of tables and plots, specifically an error histogram showing all the leaf position deviations, error RMS showing the calculated root mean square error for leaf deviations, and beam hold off and beam on plots. A vendor‐provided test plan, which moves the leaves to and from one side of the treatment field to the other covering a total distance of 140 cm, was selected for the test. To force the leaves to move at a constant speed of 2.5 cm/sec (maximum speed of HDMLC) under gravitational effect, 373 MU were delivered using 6 MV X‐rays at a dose rate of 400 MU/min in arc mode with gantry starting from 90° and stopping at 270°.^(28)^ Following the delivery, the DynaLog file was analyzed in DynaLog file viewer (DFV V7.0, Varian Medical Systems, Palo Alto, CA) for error histogram, error RMS and beam hold off and beam on plots. Loss of travel speed can result in increased beam holds or gap width errors.^(20)^

### I. Delivery of dynamic conformal arc and IMRT

Accurate delivery of dynamic conformal arc and IMRT employing both step‐and‐shoot and sliding window technique were tested for 6 MV X‐rays using manufacturer‐provided test plans and analysis of DynaLog file following recommendation from the vendor.^(28)^ In dynamic conformal arc test, a test plan (P1) consisting of a HDMLC shaped dynamic conformal field was used to deliver 160 MU at a dose rate of 400 MU/min through an arc angle of 315° to 180.5°. For step‐and‐shoot IMRT delivery test, a test plan (P2) designed to hold‐off beam exactly at three periods when leaves are in motion was exposed for 240 MU at 400 MU/min and gantry set at 0°. The performance of HDMLC in delivering sliding window IMRT was tested under gravity effect (gantry 90° and 270°) using vendor‐provided test plan (P3). The plan P3 was designed to deliver 112 MU at 400 MU/min so that the leaves move for 42 cm with a speed of 2.5 cm/sec. Following each exposure, the DynaLog file created during the beam delivery was analyzed in DynaLog file viewer for error histogram and error RMS of all the leaf position deviations, and beam hold off and beam on plots.

## III. RESULTS

### A. HDMLC alignment

**Figure 3:**
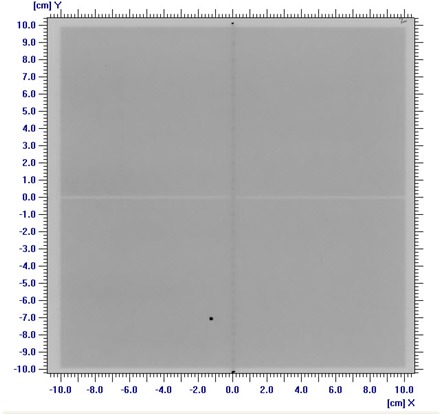
Composite image resulting from exposure using complementary alignment test pattern shown in Fig. 2. Uniform and symmetric horizontal gray line and vertical dark band demonstrate the accuracy of HDMLC alignment.

**Figure 4:**
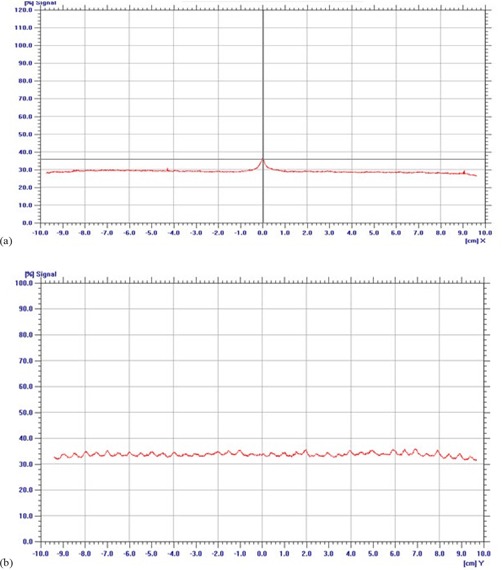
Uniform and symmetric profile (a) along the horizontal gray line of tongue‐and‐groove underdose; symmetric sinusoidal profile (b) along vertical dark band of leakage between opposing leaf end faces reveals the accuracy of HDMLC alignment.

Visual spot test of composite images acquired at zero gantry and collimator angle using complementary test pattern showed very good alignment of HDMLC leaves with isocenter plane (Fig. 3). Analysis of the same film using OmniPro IMRT software also revealed accurate alignment of HDMLC as represented by uniform and symmetric dose profile resulting from the horizontal gray line (Fig. 4(a) and symmetric sinusoidal profile from vertical dark band (Fig. 4(b). Similar results were observed for gantry 90°, 180°, and 270° with collimator at 0°. HDMLC alignment was also found unchanged at collimator 90° and 270°. The minimum simulated misalignment error spotted during visual inspection of film was 0.03 cm. This also indicates that alignment of leaves at isocenter plane along the direction of leaf motion is better than 0.03 cm at all gantry and collimator positions.

### B. HDMLC readout and radiation field congruence

The congruence of HDMLC readout and radiation field agrees within ± 0.03cm for side parallel to leaf motion, and field sizes ranging from $1\times 1\;{\text{cm}}^{2}$ to $20\times 20\;{\text{cm}}^{2}$ used in this study. For the same set of fields, the measured radiation fields along the side perpendicular to leaf motion were consistently less, by up to 0.08 cm.

### C. Radiation penumbra

**Figure 5:**
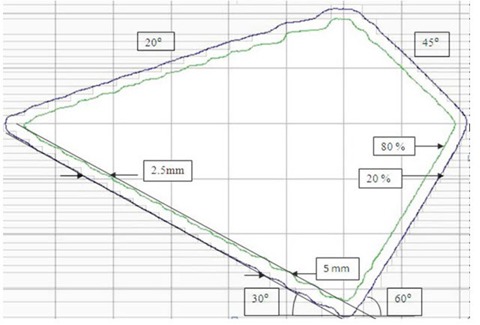
Beam's‐eye view of 80%–20% isodose curves measured for a representative irregular field.

The variation of average beam penumbra width with HDMLC‐shaped square and circular fields are presented in Table 1 for SRS6, 6, and 10 MV X‐rays. The average penumbra was least for SRS6 MV, and increases with field size and beam energy and is consistently larger along leaf motion, as compared to penumbra perpendicular to leaf motion. Mean 80% to 20% penumbra widths parallel to leaf motion were 0.24±0.05,0.37±0.12, and 0.51±0.13 cm for SRS6, 6, and 10 MV X‐rays, respectively. The corresponding values perpendicular to leaf motion were 0.21±0.02,0.29±0.07, and 0.43±0.07 cm. In the case of circular fields, the 6 MV beam penumbra parallel to leaf motion was comparable to that of corresponding square fields having similar diameter. Table 2 represents average beam penumbra at different off‐axis distances for the rectangle field size of $1\times 20\;{\text{cm}}^{2}$ and 6 MV X‐rays at 1.5 cm and 10 cm depth. The average penumbra was effectively constant over off‐axis positions of up to 12 cm, with mean value of 0.16 (± 0.01) at 1.5 cm depth and 0.38 (± 0.04)cm at 10 cm depth. Figure 5 illustrates the beam's‐eye view of 80% and 20% isodose curves measured for a representative irregular field. It also shows the steep angle between diagonal straight field edge and direction of leaf motion. The effective penumbra along the straight diagonal field edge increases with the increase of leaf width and decrease of steep diagonal angle. Table 3 represents the mean effective penumbra corresponding to 0.25 cm and 0.5 cm leaf width along the diagonal straight field edge of various steep angles. The mean effective penumbra corresponding to 0.25 cm leaf width increases marginally from 0.3 cm at 70° steep diagonal angle to 0.35 cm at 20°. The corresponding increase in effective penumbra for 0.5 cm leaf width was higher at 0.32 cm to 0.56 cm.

**Table 1:** Average radiation penumbra (80%–20%) of different HDMLC defined square and circular fields measured at $d_{\text{max}}$ for SRS6, 6, and 10 MV X‐rays.

	*Average Penumbra in cm for Square and Circular Fields*							
	*Parallel to Leaf Motion*			*Perpendicular to Leaf Motion*				
*Field Size* $\left({\text{cm}}^{2}\right)$	*SRS6 MV*	*6 MV*	*10 MV*	*SRS6 MV*	*6 MV*	*10 MV*	*Circular Field Diameter (cm)*	*Parallel to Leaf Motion 6 MV*
1×1	0.20	0.27	0.33	0.20	0.24	0.32	1	0.25
2×2	0.23	0.29	0.41	0.20	0.27	0.38	2	0.30
3×3	0.23	0.31	0.43	0.19	0.25	0.38	3	0.31
4×4	0.24	0.33	0.49	0.20	0.27	0.43	4	0.33
5×5	0.25	0.36	0.52	0.21	0.29	0.43	5	0.34
10×10	0.26	0.42	0.63	0.21	0.30	0.49	10	0.38
15×15	0.29	0.47	0.62	0.23	0.34	0.48	15	0.40
20×20		0.49	0.64		0.36	0.50		

**Table 2:** Average radiation penumbra (80%–20%) width of $1\times 20\;{\text{cm}}^{2}$ field measured at different off‐axis distances for 6 MV X‐rays at 1.5 cm and 10 cm depth.

	*Average Beam Penumbra (SD) in cm at Off‐axis Distances*			
*Depth of Measurement*	*0 cm*	*4 cm*	*8 cm*	*12 cm*
1.5 cm	0.17 (0.01)	0.16 (0.01)	0.16 (0.01)	0.16 (0.01)
10 cm	0.37 (0.03)	0.39 (0.04)	0.38 (0.03)	0.38 (0.03)

**Table 3:** Mean effective penumbra corresponding to 0.25 cm and 0.5 cm leaf width along the diagonal straight field edge of various steep angles.

	*Mean Effective Penumbra in cm Corresponding to Leaf Width of:*	
*Steep Angle*	*0.25 cm*	*0.5 cm*
20°	0.35	0.56
30°	0.34	0.50
40°	0.34	0.46
45°	0.33	0.43
50°	0.32	0.41
60°	0.30	0.37
70°	0.30	0.32

### D. Leaf position and gap width in DMLC mode

A visual spot check of modified Picket Fence test film shows very distinct and uniform dark bands separated by a fixed distance, which is in accordance to planned leaf pattern (Fig. 6(a). Quantitative analysis of the film revealed overestimation in band width with an average FWHM of 0.18 cm (SD=0.01), but shows very good agreement in interband separation with average peak‐to‐peak distance of 1.99 cm (SD=0.03) (Fig. 6(b). A minimum simulated error of 0.03 cm in 0.5 cm leaves was detected in a visual spot check, as compared to 0.05 cm in 0.25 cm leaves. The output factor of DMLC fields with 0.2, 0.5 and 1 cm sweeping filed width were 0.05, 0.09 and 0.14 for both 6 and 10 MV X‐rays. DMLC output factor for 0.5 cm sweeping field width remains within ± 1% for 6 MV and 0.5% for 10 MV X‐rays at all gantry positions. The long‐term reproducibility of the leaf position readout observed during 14 months was within 0.05 cm. Figure 7 shows the variation of DMLC output factor for 6 and 10 MV X‐rays over a period of 14 months. DMLC field output factor was reproducible within ± 0.5% for both 6 and 10 MV X‐rays during this period, except on a few occasions where a variation of around ± 1.0% was observed in the 0.2 cm sweeping gap width and 10 MV X‐rays only.

**Figure 6(a):**
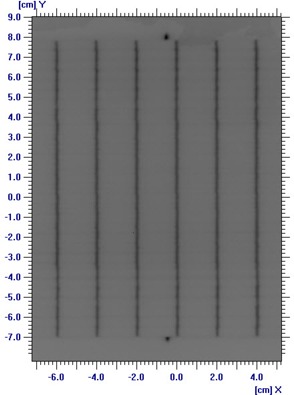
Image of the modified Picket Fence test for leaf position and gap width accuracy test.

**Figure 6(b):**
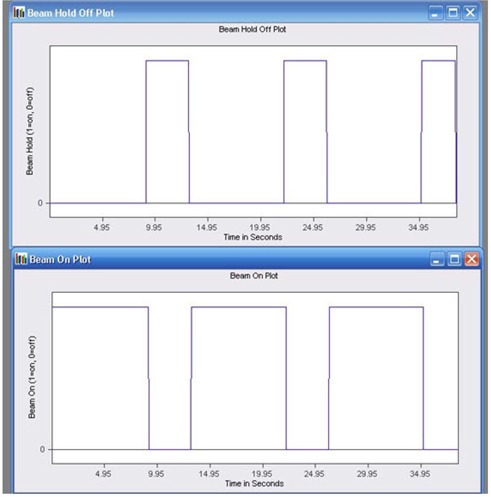
Profile taken at the middle of the central leaf pair and along the direction of leaf motion. The band width and interband separation are represented by FWHM of each profile and peak‐to‐peak distance of adjacent profiles.

**Figure 7:**
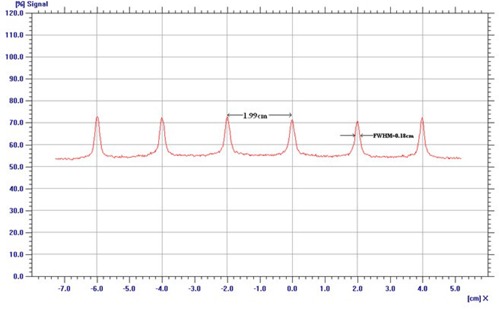
Variation of normalized DMLC field output factor for 6 and 10 MV X‐rays over a period of 14 months. DMLC fields had different sweeping field width of 0.2 cm. 0.5 cm and 1 cm.

### E. Static and dynamic leaf shift

**Figure 8:**
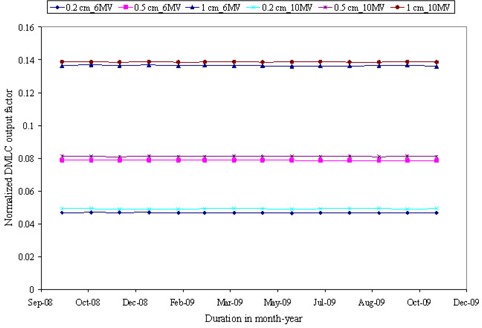
Variation of measured dose free from leakage with sweeping gap width for 6, 6SRS and 10 MV X‐rays for the calculation of dynamic leaf shift.

The static leaf shift was within 0.03 cm for all beam energies. Figure 8 represents the variation of dose free from HDMLC leakage for DMLC fields of various gap widths and for all three photon energies. Dynamic leaf shift for 10 MV X‐rays was slightly larger at 0.044 cm as compared to 0.039 cm of 6 MV and SRS 6MV.

### F. Tongue‐and‐groove effect

**Figure 9:**
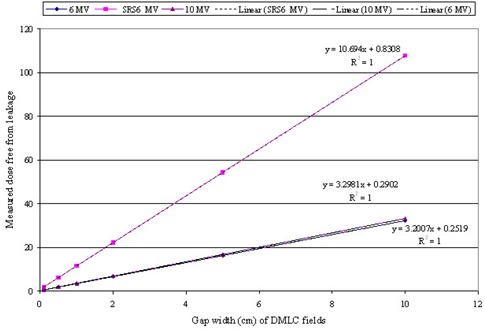
Dose depression due to tongue‐and‐groove effect of HDMLC for 6 and 10 MV X‐rays.

The normalized profile along the match‐line and perpendicular to leaf motion is shown in Fig. 9 for 6 and 10 MV X‐rays. The dose reduction in the overlap region of leaf faces produced by tongue‐and‐groove design was 24% and 19% for 6 and 10 MV X‐rays, and the corresponding tongue‐and‐groove size measured as FWHM was 0.17 and 0.2 cm.

### G. Leaf transmission and leakage

**Figure 10:**
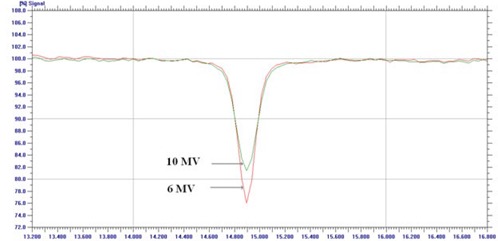
Transmission through single bank leaves that abut with opposing leaves at 5 cm off‐axis.

Average leakage through HDMLC with jaws open was 1.09%, 1.14% and 1.34% for SRS6 MV, 6MV and 10 MV X‐rays, respectively. The corresponding values with jaws closed were 0.01% for all the energies. Figure 10 represents the relative dose profile obtained across closed HDMLC for 6 MV X‐rays. The transmission through the leaves had maximum and minimum value of 1.52% and 0.86%, respectively.

### H. Leaf travel speed test

**Figure 11:**
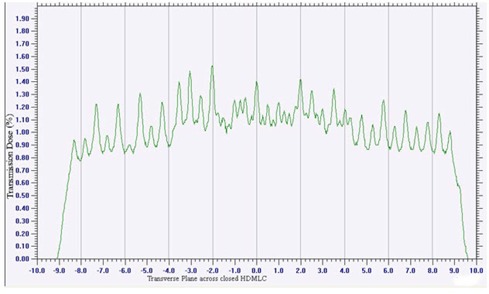
Error histogram and error RMS resulting from the arc dynamic leaf speed test.

Figure 11 shows the error histogram and error RMS resulting from the arc dynamic leaf speed test as viewed in the DynaLog file viewer application. The error histogram showed that at least 95% or more of the error counts have misplacements < 0.2cm and there is no error count with misplacements > 0.3cm. The maximum RMS error was 0.14 cm.

### I. Delivery of dynamic conformal arc and IMRT

**Figure 12:**
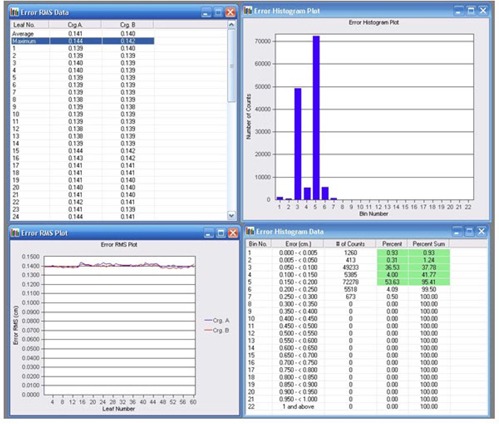
Beam‐hold‐off versus time and Beam On versus time plot from step and shoot IMRT test plan.

Analysis of DynaLog file for the dynamic conformal arc test showed that at least 95% or more of the error counts are within a misplacement of 0.05 cm, with a maximum RMS error of 0.02 cm. Figure 12 shows the Beam On vs. Time and Beam‐hold‐off vs. Time plot from step‐and‐shoot IMRT test plan, as viewed in DFV application. The time synchronization of three periods of Beam On and three periods of Beam‐hold‐off directly opposite to the beam‐on plot reveal successful delivery of step‐and‐shoot IMRT test plan. The delivery of sliding window IMRT test plans at both gantry angles of 90° and 270° showed at least 95% or more of the error counts within misplacements of 0.15 cm, with maximum RMS error value calculated at 0.13 cm.

## IV. DISCUSSION

Alignment of MLC leaves affects the spatial uncertainty between the peripheral extent of radiation beams and the perimeter of the target volume.^(^ ^10^ ^,^ ^11^ ^,^ ^13^ ^)^ The alignment test during HDMLC calibration using vendor‐provided tools and software does not quantify the magnitude of alignment accuracy. Film verification test showed HDMLC alignment accuracy better than 0.03 cm, which is the minimum detectable misalignment on film. The horizontal gray line and vertical dark band appearing on the HDMLC alignment test film was due to the tongue‐and‐groove underdose and leaf end transmission. Any misalignment will lead to nonuniform intensity of horizontal gray line which, in turn, will show asymmetry in horizontal profile and irregularity in sinusoidal profile shape. The effect of gravity on HDMLC alignment was not observed, as the alignment accuracy was unchanged at all gantry rotations. Simulated HDMLC misalignment test is useful in indirectly quantifying alignment accuracy and also to understand the strength of the test tools for subsequent routine QA and setting of action level. While interpretation of such tests is subjective, it gives a good idea about the alignment accuracy of HDMLC. This test can also be adopted as a quick QA check using an online verification tool such as a high‐resolution electronic portal imaging device (EPID).

Agreement between MLC readout and radiation field is important in high‐precession radiotherapy such as conformal therapy with small fields, SRS and IMRT. In the case of rounded end MLCs, the agreement between the digital MLC position readout and the light field or radiation field edges is achieved through calibration software and calibration methods. Several authors^(^ ^2^ ^,^ ^12^ ^,^ ^16^ ^,^ ^18^ ^)^ have reported a difference between leaf position readout and radiation field ranging from 0.05 to 0.12 cm per side. Graves et al.^(18)^ illustrated significant improvement in MLC readout and radiation field congruence accuracy close to 0.03 cm when an independent automatic correction software was incorporated with the Varian MLC controller system. The new calibration method and software incorporated in HDMLC controller resulted very good readout and radiation field agreement, well within 0.03 cm. Relatively lesser value of radiation field consistently observed in all field sizes along the direction perpendicular to leaf motion was similar to that observe for upper jaws, and may be due to the position of focal spot of the optical field.

The observed increase in penumbra with field size, beam energy, depth of measurement, and along leaf motion is in agreement with other studies.^(^ ^6^ ^–^ ^8^ ^,^ ^21^ ^)^ The smaller penumbra observed for SRS6 MV as compared to nominal 6 MV in our study could be due to the variation in focal spot size of the target and flattening filter. Radiation penumbra measured for 6MV X‐rays at $d_{\text{max}}$ and parallel to leaf motion of square fields was comparable to the value reported by Chang et al.^(25)^ for similar HDMLC. The penumbra from SRS6 MV was not available for comparison. Our measured penumbra for 6 MV X‐rays and field size up to $10\times 10\;{\text{cm}}^{2},0.27-0.42\;\text{cm}$ parallel and 0.24–0.30 cm perpendicular to leaf motion were slightly larger as compare to 0.2 to 0.35 cm reported by others^(^ ^6^ ^–^ ^8^ ^)^ for the BrainLAB micromultileaf collimator. This could be primarily due to the variation in MLC leaf width, leaf end and face design, and its integration to the collimator system. Cosgrove et al.^(6)^ reported mean penumbra of 0.26±0.01 cm (parallel) and 0.25±0.01 cm (perpendicular) to leaf motion for 6 MV X‐rays and field size from $2\times 2\;{\text{cm}}^{2}$ to $10\times 10\;{\text{cm}}^{2}$ for BrainLAB mMLC attached as an add‐on device to a Varian linac. Though HDMLC has a thinner leaf width than the BrainLAB mMLC, a slightly larger mean penumbra of 0.34±0.02 cm (parallel) and 0.28±0.02 cm (perpendicular) to leaf motion was observed for the same field size and beam energy. This small increase in penumbra could be due to a larger isocentric clearance of about 45 cm in Novalis Tx, as compared to 31 cm in BrainLAB mMLC integrated to the linac.^(6)^ However, the penumbra parallel and perpendicular to leaf motion from the radiosurgery beam (SRS6 MV) is much sharper, with a mean value of 0.24±0.02 cm and 0.20±0.01 cm, respectively. The consistency of the beam penumbra observed for asymmetric fields in off‐axis positions is a desirable characteristic as it simplifies modeling of dose distribution from this MLC by any treatment planning computer without additional beam data. The broadening of penumbra with increase in leaf width and decrease in steeped angle is in agreement with other studies.^(^ ^6^ ^,^ ^9^ ^,^ ^22^ ^)^ Our effective penumbra value for 0.25 cm and 0.5 cm leaf width at various steep angles is similar to minimum and maximum value reported by Cosgrove et al.^(6)^ In that paper, the steeping angle was defined as being between the straight diagonal edge of the field and the line perpendicular to the direction of HDMLC motion. Our measured minimum 0.33±0.03 mm and maximum 0.43±0.13 effective penumbra at 45° steep angle is comparable to 0.33±0.03 and 0.50±0.03 reported by Cosgrove et al.^(6)^ Liu at al.^(9)^ reported an effective penumbra of 0.5, 0.3 and 0.2 cm for the Varian millennium 120 MLC, dual‐layer mMLC and cerrobend block, which is also in agreement with our results.

In IMRT and dynamic SRS, leaf gap width and positional accuracy of MLC affect the dosimetric accuracy and spatial dose distribution relative to the anatomy. A positional error of 0.1 cm in a 1 cm sliding window can produce dosimetric error as high as 10%.^(12)^ In Varian MLCs, precision of the leaf position and gap depends upon the gap calibration methods and proprietary controller software system which interactively monitor the leaf motion. Graves et al.^(18)^ have reported calibration methods of previous versions of Varian MLCs and illustrated possible corrective measures to improve precision. While the controller hardware and general operational concept of HDMLC is similar to the millennium 120 MLC, the calibration method of HDMLC and controller software (V7.2) is different. Leaf positioning accuracy and repeatability of HDMLC is well within the tolerance limit of 0.1 cm recommended in AAPM TG 142.^(23)^ LoSasso et al.^(12)^ reported that a 3% change in the normalized DMLC output of 0.5 cm sweeping gap width corresponds to a 0.02 cm change in the gap width. A maximum variation of 1% observed in the normalized DMLC output of 0.5 cm sweeping gap as compared to corresponding measurement taken at zero gantry and collimator angle indicate that leaf positioning accuracy of HDMLC is better than 0.02 cm, and is found to be unaffected by change in gantry and collimator rotation. The variation of the normalized DMLC output of 0.5 cm sweeping gap width observed over a period of 14 months was also within 0.5% for both energies, indicating that HDMLC positioning accuracy was reproducible well within 0.02 cm.

The dynamic leaf shift observed in our study was comparable to that of Cheng et al.^(25)^ but static leaf shift was not available for comparison. The average HDMLC transmission was also comparable with the findings of the Cheng et al. study, except for the higher energy where the authors have reported same average transmission from 6 and 15 MV X‐rays. The average transmission from HDMLC for 6 MV X‐ray is less when compared to 1.9% of BrainLAB micromultileaf collimator^(6)^ and 1.5% of millennium 120 MLC.^(29)^ The tongue‐and‐groove underdose was marginally larger as compared to 21% reported from millennium MLC.^(29)^

The accuracy of the information contained in the Varian MLC log files have been validated using film measurements, diode array measurements, and portal imaging.^(^ ^30^ ^–^ ^32^ ^)^ Few studies have demonstrated the application of DynaLog file analysis for the commissioning and quality assurance of IMRT.^(^ ^24^ ^,^ ^27^ ^)^ Based on previous studies,^(^ ^24^ ^,^ ^27^ ^,^ ^30^ ^–^ ^32^ ^)^ the recent AAPM TG142report^(23)^ also recommended the use of DynaLog files for machine QA and set acceptable criteria. Based on the recommendation, the error histogram is deemed acceptable if 95% of the leaf deviations are less than 0.35 cm and the maximum error RMS for either carriage is less than 0.35 cm. The analysis of the DynaLog file generated during arc dynamic leaf speed test, delivery of dynamic conformal arc, step‐and‐shoot and sliding window IMRT under complex conditions, shows that error histogram, maximum error RMS, beam hold off and beam on plot well within the acceptable limit.

## V. CONCLUSIONS

The high‐definition multileaf collimator (HDMLC) control by new software showed leaf positioning accuracy better than 0.03 cm at all gantry and collimator positions; these results were reproducible within 0.05 cm and 0.5% in DMLC output over a period of 14 months. It also showed less leakage, and a small and more constant penumbra across the field. Among the three photon energies, the radiosurgery beam (SRS6MV) showed sharpest penumbra. HDMLC successfully passed the dynamic conformal arc and IMRT delivery test specified in AAPMTG 142. The favorable physical and dosimetric characteristics of HDMLC and its ability to shape small irregular targets using finer leaf while providing large field size and isocentric clearance, demonstrated the potential of HDMLC on Novalis Tx for small‐field high‐dose resolution SRS and IMRT.
